# Pelota interacts with HAX1, EIF3G and SRPX and the resulting protein complexes are associated with the actin cytoskeleton

**DOI:** 10.1186/1471-2121-11-28

**Published:** 2010-04-20

**Authors:** Ozanna Burnicka-Turek, Aleksandra Kata, Byambajav Buyandelger, Linda Ebermann, Nadine Kramann, Peter Burfeind, Sigrid Hoyer-Fender, Wolfgang Engel, Ibrahim M Adham

**Affiliations:** 1 Institute of Human Genetics, Georg-August-University, Göttingen, Germany; 2 Department of Developmental Biology, GZMB, Georg-August-University, Göttingen, Germany

## Abstract

**Background:**

Pelota (PELO) is an evolutionary conserved protein, which has been reported to be involved in the regulation of cell proliferation and stem cell self-renewal. Recent studies revealed the essential role of PELO in the No-Go mRNA decay, by which mRNA with translational stall are endonucleotically cleaved and degraded. Further, PELO-deficient mice die early during gastrulation due to defects in cell proliferation and/or differentiation.

**Results:**

We show here that PELO is associated with actin microfilaments of mammalian cells. Overexpression of human PELO in Hep2G cells had prominent effect on cell growth, cytoskeleton organization and cell spreading. To find proteins interacting with PELO, full-length human PELO cDNA was used as a bait in a yeast two-hybrid screening assay. Partial sequences of HAX1, EIF3G and SRPX protein were identified as PELO-interacting partners from the screening. The interactions between PELO and HAX1, EIF3G and SRPX were confirmed *in vitro *by GST pull-down assays and *in vivo *by co-immunoprecipitation. Furthermore, the PELO interaction domain was mapped to residues 268-385 containing the c-terminal and acidic tail domain. By bimolecular fluorescence complementation assay (BiFC), we found that protein complexes resulting from the interactions between PELO and either HAX1, EIF3G or SRPX were mainly localized to cytoskeletal filaments.

**Conclusion:**

We could show that PELO is subcellularly localized at the actin cytoskeleton, interacts with HAX1, EIF3G and SRPX proteins and that this interaction occurs at the cytoskeleton. Binding of PELO to cytoskeleton-associated proteins may facilitate PELO to detect and degrade aberrant mRNAs, at which the ribosome is stalled during translation.

## Background

The Pelota gene (*Pelo*) encodes an evolutionary conserved protein which has been identified in various species [1-3]. The protein contains an RNA binding domain similar to that found in members of the eukaryotic release factor 1 (eRF1) family, which are involved in terminal stop of protein synthesis [4].

The biological role of PELO was originally identified in *D. melanogaster*. In male mutants, mitosis during germ cell development is normally progressed, but the cell cycle of the first meiotic division is arrested in late prophase stage. On the contrary, during oogenesis only mitotic division is affected. Moreover, the eye of mutant flies has disordered ommatidial array and the orientation of bristles suggests the impairment of planar cell polarity during eye development [5]. The role of PELO in meiotic and mitotic division was also confirmed in *S. cerevisiae*, where the deletion of the *Pelo *orthologue gene DOM34 causes growth retardation and defective sporulation. The decrease of polyribosomal and increase of free ribosomal fraction in dom34? mutants suggest a participation of PELO in the machinery of protein synthesis or in the regulation of mRNA translation [4].

Translational control of eukaryotic gene expression plays an essential role in the development and differentiation of cells and provides an important checkpoint for cell growth and differentiation. Deletion of the *Pelo *gene in mice revealed that PELO-deficient embryos failed to develop after implantation. Culture of blastocyts *in vitro *demonstrated the failure of mitotically active inner cell mass (ICM) of *Pelo*^-/- ^blastocysts to expand in growth. These results suggest that PELO is essential for regulation of the cell cycle during gastrulation [6]. Another interpretation for the failure of the ICM to proliferate might be due to the fact that stem cells of *Pelo*^-/- ^embryos fail to self-renewal. The role of PELO in control of germ stem cell (GSC) self-renewal has already been shown in *D. melanogaster *[7]

To further elucidate the possible function of PELO, we searched for proteins that bind to PELO. We performed a yeast two-hybrid screen using PELO as a bait. Several positive clones including HAX1, EIF3G and SRPX were isolated. Using GST pull-down assay and co-immunoprecipitation, we confirmed the specific interactions of PELO with the putative interacting proteins and found that the c-terminal and acidic tail domains are responsible for the interactions with partner proteins. To further support the specificity of interactions between PELO and its putative partners and to determine the subcellular localization of the interacting proteins, we performed bimolecular fluorescence complementation assay (BiFC), and observed that protein complexes resulting from the interactions between PELO and either HAX1, EIF3G or SRPX are associated with the cytoskeleton.

## Results

### PELO is subcellularly localized at the actin microfilaments

**Figure 1:**
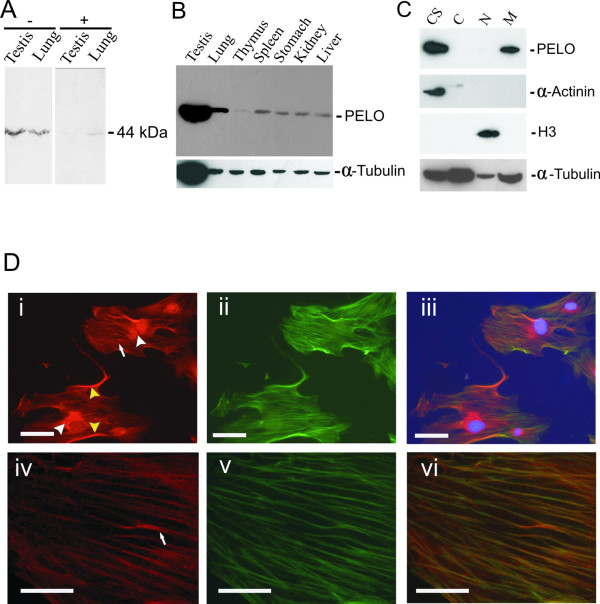
**Subcellular localization of PELO at the cytoskeleton**. **A**, Competition assay to determine the specificity of PELO antibodies. Blots with testis and lung protein extracts were incubated with anti-PELO antibodies in presence (+) or absence (-) of the GST-PELO fusion protein. **B**, Immunoblot with cellular extracts from different tissues was probed with anti-PELO antibodies. **C**, PELO is distributed in cytoskeletal and membrane fractions. Immunoblots with cellular extracts from different tissues were probed with anti-PELO antibodies. Blots containing samples of cytoskeletal (CS), cytoplasmic (C), nuclear (N) and membrane (M) fractions from HeLa cells were subsequently probed with polyclonal anti-PELO, monoclonal anti-α-actinin, anti-phospho H3 histone and anti-α-tubulin. **D**, Co-localization of the PELO antigen and actin stress fibers. Fibroblasts were immunolabeled with PELO antibodies (i, iv) or FITC-phalloidin (ii, v). PELO immunostaining was localized at the cytoskeleton (white arrows), at some regions of plasma membrane (yellow arrowhead), and near nuclear membranes (white arrowhead). The merged images (iii, vi) show that PELO largely co-localized with actin-stress fibers. Scale bars = 20 μm.

RNA analysis revealed ubiquitous expression of *Pelo *[2,3]. To determine the subcellular localization, anti-PELO antibodies were generated against a GST-PELO fusion protein containing the full-length human amino acid sequence. Affinity-purified GST-PELO antibodies predominantly detected a 44 kDa protein band (Figure 1A, left panel), which is consistent with the predicted molecular mass of PELO. Detection of the 44 kDa protein was greatly reduced or even not found after preabsorption of the antibody with the antigen, to which it was, raised (Figure 1A, right panel). PELO expression was then examined by Western blot analysis and immunohistochemistry. Western blot analysis showed that anti-PELO recognizes the 44 kDa protein band in a wide variety of mammalian cells and tissues (Figure 1B). The subcellular localization of PELO was firstly studied by western blot analysis, we used two different biochemical fractionation techniques as described in Methods part to produce cytoskeletal, cytoplasmic, nuclear and membrane extracts. Purity of the fractions was assessed by examining each fraction for the presence or absence of α-actinin, a cytoskeletal marker, and phospho H3 histone, a nuclear marker. As expected, α-actinin and phospho-histone H3 were only found in the cytoskeletal and nuclear fractions, respectively. PELO was exclusively present in the cytoskeletal and in the membrane fractions (Figure 1C). The proportion of PELO in the cytoskeletal fraction was higher than that in the membrane fraction. To confirm the association of PELO with the cytoskeleton, cultured primary fibroblast was immunofluorescently stained with anti-PELO antibody. A fibrillar staining pattern was observed in the cytoplasm. PELO also localized at some regions of plasma membrane and near the nuclear membrane presumably to the endoplasmic reticulum (Figure 1D, panel i, iv). To study whether PELO is associated with the actin cytoskeleton, cells were probed with FITC-labelled phalloidin that stains polymerized F-actin (Figure 1D, panel ii, v). Overlay images demonstrate co-localization of F-actin and PELO (Figure 1D, panel iii, vi), that implies that PELO is associated with actin microfilaments. This result is consistent with that obtained by Western blot analysis.

### PELO overexpression disrupts actin stress fibers and reduces cell growth and spreading

**Figure 2:**
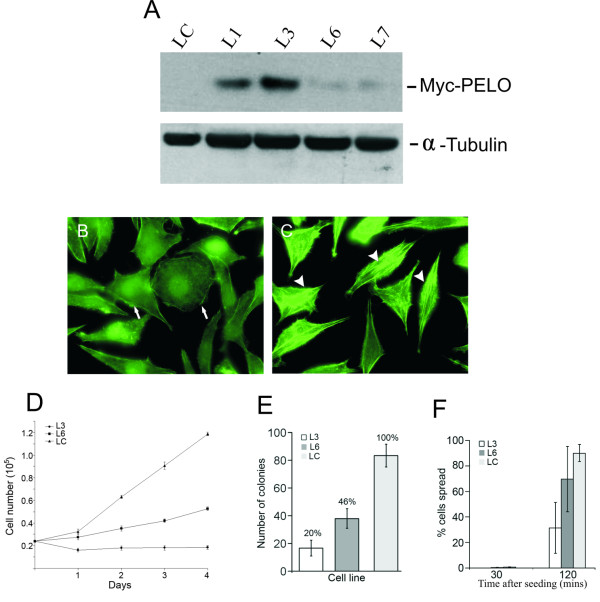
**Overexpression of PELO affects actin stress fibers, cell growth and spreading in stable PELO-overexpressing cell lines**. **A**, Hep2G cells were transfected with Myc-PELO fusion constructs or empty vector. Protein extracts from stable control (LC) and Myc-PELO expressing cell lines (L1, L3, L6 and L7) were analyzed by immunoblotting with monoclonal anti-Myc tag antibody. The membrane was subsequently reprobed with α-tubulin antibody. **B-C**, Cells of Myc-PELO-overexpressing L3 (B) and L6 (C) cell lines were stained with FITC-phalloidin. Cell showed diffusely cytoplasmic actin are labelled with arrows and that containing stress actin with arrowheads. **D**, *In vitro *growth properties of control and PELO-overexpressing cells. Growth curves of mock-transfected Hep2G (LC), and PELO-transfected clone L3 and L6. **E**, Growth of the same cell lines in soft agar. 0.2 × 10^4 ^cells were plated per dish. 100% (LC) = 4.4 × 10^2 ^colonies. The data points represent the means ± SD of three independent experiments. **F**, Control cells (LC) as well as L3 and L6 that expressed PELO at low and high levels, respectively, were seeded onto fibronectin-coated slides for 30 and 120 min. Cells were then counted and scored for spreading. White bars represent L3, black bars L6 and grey bars LC cells. Results are presented as percentage of cells that adapted spread morphology at 30 and 120 min after seeding. 100 cells per well were counted in three separate experiments. Data represent means ± SD.

To determine the effect of PELO overexpression, Hep2G cells were transfected with either Myc-tagged PELO or an empty pcDNA vector as a control and stable transfected clones were selected. Expression and specificity of the transfected construct were confirmed by Western blot analysis using monoclonal antibody against the c-Myc epitope. As shown in Figure 2A, the anti-Myc antibody specifically recognizes a 46 kDa Myc-PELO fusion protein in extracts of recombinant clones, but not in control cell line. We then used the clones L3 and L6, which exhibit higher and lower levels of Myc-PELO expression, respectively, in further experiments. To determine cytoskeleton organization in Myc-PELO-overexpressing cell lines, cells were stained with FITC-phalloidin. In cells expressing high levels of Myc-PELO (L3), no stress fibers were detected by phalloidin staining (Figure 2B). However, F-actin is still present in these cells as shown by diffuse cytoplasmic phalloidin-FITC labelling (Figure 2B). In contrast, actin stress fibers were intact in low Myc-PELO-expressing cells (L6) (Figure 2C). These results suggest that expression of PELO at high levels results in disassembly of stress actin.

Proliferation assays showed a significant reduction in growth rate of stable Myc-PELO-overexpressing cell lines L3 and L6 as compared to control cells (Figure 2D). The level of reduction in growth rate between both Myc-PELO-overexpressing cell lines correlated with the expression level of Myc-PELO (Figure 2A). Anchorage-independent growth is a generally accepted indicator of the transformed phenotype *in vitro*. To investigate whether the overexpression of PELO alters the anchorage-independent growth ability of HepG2 hepatoma cells, we compared the ability of L3 and L6 to growth in soft agar. As shown in Figure 2E, the number of colonies in L3 and L6 cell lines was significantly lower than those of mock-transfected HepG2 cells (LC). The anchorage-independent growth capacity was also correlated with the level of PELO protein in each cell line. To investigate whether the disruption of actin stress fibers in Myc-PELO-overexpressing cells affects the spreading behaviour, cells were seeded onto fibronectin-coated plates. At 30 min after seeding, the majority of Myc-PELO-overexpressing and control cells had not spread and exhibited a round morphology. At 2 h after seeding, 34% and 70% of L3 and L6 cells were spread, respectively, compared with 90% of control cells (Figure 2F). Taken together, these results indicate that Myc-PELO-overexpression affects cell proliferation and spreading following cell adhesion.

### PELO interacts with HAX1, EIF3G and SRPX

To determine the molecular basis of PELO association with the cytoskeleton, a yeast two-hybrid screen was performed to identify PELO-interacting proteins. As a bait, we cloned full length PELO into the screening vector. With this screening, several putative interacting partners for PELO were identified. Among them, two clones contain a cDNA sequence for HAX1, and two clones coding a sequence of EIF3G and SRPX. The identified cDNA fragments contained the partial coding sequences of HAX1 (amino acid residues 176-279), EIF3G (amino acid residues 1-320) and SRPX (amino acid residues 265-464). Several control experiments were performed to assess the fidelity of these interactions in yeast. Neither the bait nor HAX1, EIF3G or SRPX vectors autonomously activated the reporter genes in the yeast two-hybrid system, when grown in the appropriated nutrient-deficient media (minus amino acid histidine, adenine and tryptophan with addition of 5 mM 3-amino-1, 2, 4 triazole) (data not shown).

**Figure 3:**
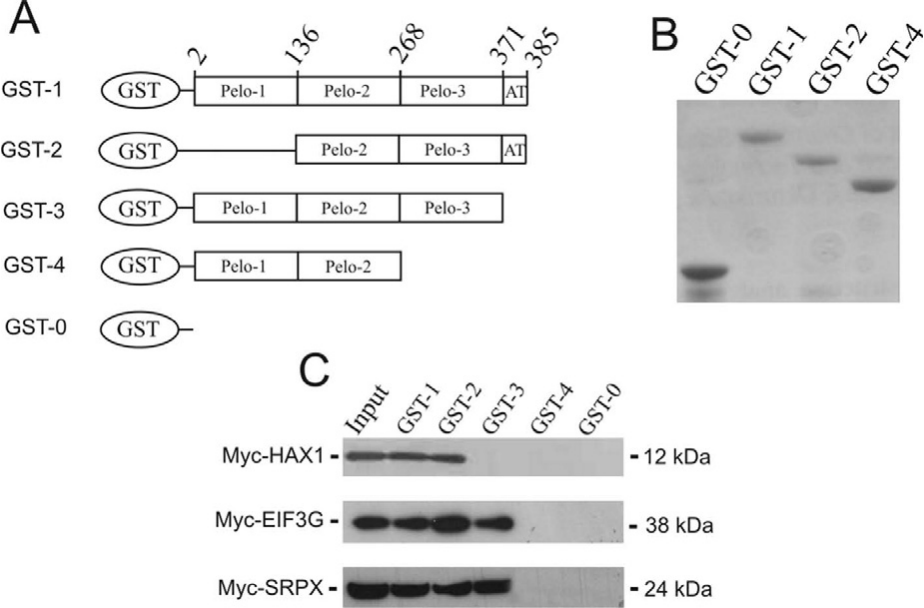
**The acidic tail (AT, residues 371-385), and c-terminal (Pelo-1, residues 268-371) region of PELO is required for binding to HAX1, and EIF3G and SPRX, respectively**. **A**, To determine the regions of PELO that are required for its association with HAX1, EIF3G and SRPX, four GST-PELO fusion proteins were generated (GST-1, GST-2, GST-3 and GST-4) and tested for their ability to bind in GST pull-down assays. GST alone (GST-0) was used as control in the assay. **B**, Recombinant GST-PELO fusion proteins were subjected to SDS-PAGE and stained with Coomassie blue. **C**, Equivalent amounts of GST-PELO and GST-0 proteins bound to glutathione-matrices were subsequently incubated with extracts of HeLa cells, which were transfected with either Myc-tagged HAX1, EIF3G or SRPX constructs. Western blots containing the GST pull-down assays were incubated with monoclonal anti-Myc antibodies. Immunoblot analysis revealed that GST-1 and GST-2, but not GST-3, GST-4 and GST-0 bound to Myc-HAX1 protein. Except GST-4 and GST-0, all other GST-PELO fusion proteins exhibit specific association to Myc-EIF3G and Myc-SRPX.

To identify and verify the region of PELO responsible for specific interactions with HAX1, EIF3G and SRPX, several GST-PELO fusion proteins were generated containing deletions of different PELO domains (Figure 3A, B). Next, the binding activity of the different GST-PELO fusion proteins with the putative interacting proteins was tested in a GST pull-down assay. Cells lysates from HeLa cells transiently transfected with Myc-tagged HAX1, EIF3G or SRPX expression vectors were incubated with different GST-PELO fusion proteins bound to glutathione-sepharose beads. After extensive washing, proteins bound to beads were immunoblotted with monoclonal anti-Myc antibody. As shown in the top panel of Figure 3C, GST-1 and GST-2 fusion proteins, but not GST-3, GST-4 and GST-0 interact with the 12 kDa Myc-HAX1 (Figure 3C). Deletion of the acidic tail domain (AT) of PELO (residues 271-285) in both GST-3 and GST-4 fusion proteins demonstrates that the AT domain of PELO is responsible for binding to HAX1. In further pull-down assays with protein extracts of HeLa cells transfected with either Myc-tagged EIF3G or SRPX constructs, GST-1, GST-2 and GST-3, but not GST-4 and GST-0 exhibited strong association to the 38 kDa Myc-EIF3G (Figure 3C, middle panel) and 24 kDa Myc-SRPX, respectively (Figure 3C, bottom panel). These results indicate that the c-terminal domain of PELO (residues 268-370) is essential for binding to EIF3G and SRPX *in vitro*. Taken together, these results confirm the interactions of PELO with HAX1, EIF3G and SPRX, which were found as interaction proteins of PELO in a yeast two-hybrid assay.

**Figure 4:**
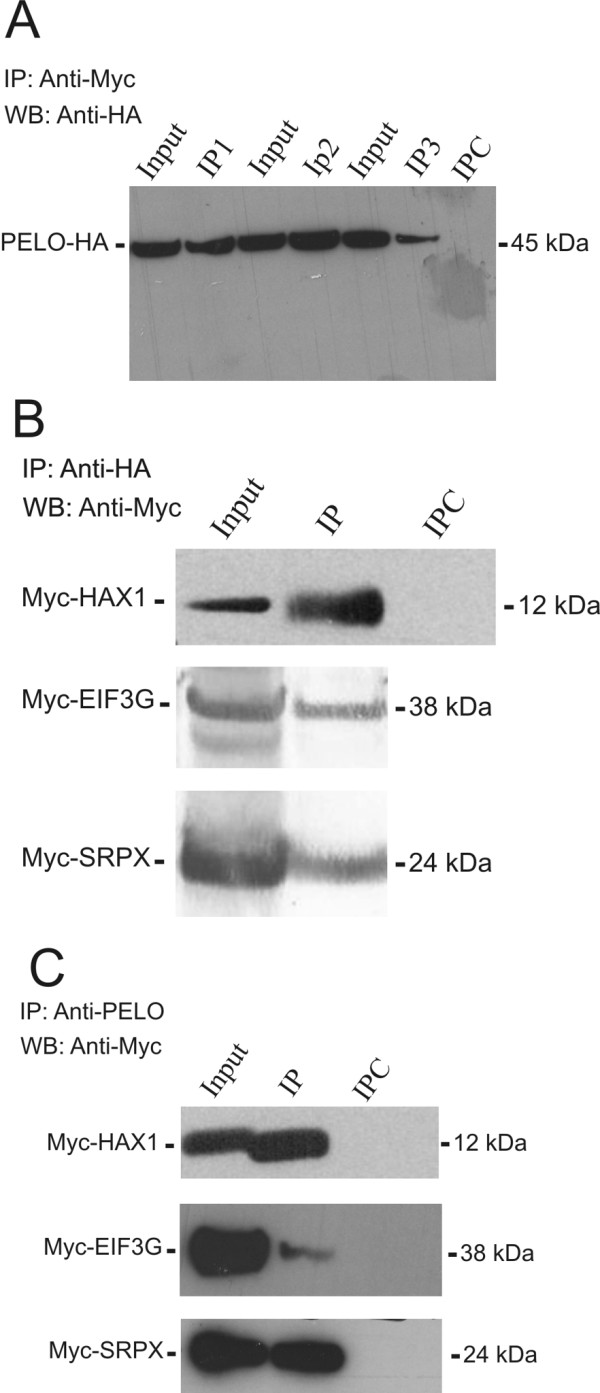
**PELO interacts with HAX1, EIF3G and SRPX *in vivo***. **A**, Total protein from HeLa cells, which are co-transfected with HA-tagged PELO and either Myc-tagged HAX1, EIF3G or SRPX constructs, were precipitated with monoclonal anti-Myc antibodies. The precipitates of co-transfected cells with PELO-HA:Myc-HAX1 (IP1), PELO-HA:Myc-EIF3G (IP2) and PELO-HA:Myc-SRPX (IP3) were probed in immunoblot analysis with polyclonal anti-HA antibodies. IPC, control co-immunoprecipitation (coIP) assay using anti-mouse IgG serum. **B-C**, In reciprocal coIP experiments, cells lysates from co-transfected HeLa cells were precipitated with either polyclonal anti-HA (B) or anti-PELO antibodies (C). Precipitated proteins (IP) were detected with anti-Myc antibodies. IPC, control coIP experiments using anti-rabbit IgG serum.

The interactions of PELO with the putative interacting proteins were further studied in HeLa cells, which were transiently co-transfected with HA-tagged PELO (PELO-HA) expression vectors and either Myc-tagged HAX1, EIF3G or SRPX constructs. Cell lysates were subjected to immunoprecipitation with a monoclonal anti-Myc antibody or control mouse IgG, followed by western blot analysis with a polyclonal anti-HA antibody. A single polypeptide band of 45 kDa in size, consistent with the molecular mass of PELO-HA, was detected in input lysates and in all immunoprecipitated assays with anti-HA, but not in control mouse IgG-immunoprecipitates (Figure 4A). In the opposite experiments, lysates of co-transfected HeLa cells with PELO-HA and either Myc-tagged HAX1, EIF3G or SRPX construct were immunoprecipitated with either HA-tag or PELO antibodies. The precipitates were then immunoblotted and probed with monoclonal Myc-tag antibodies. Single polypeptide bands of 12 kDa, 38 kDa and 24 kDa in size, consistent with the expected molecular masses of cloned Myc-HAX1, Myc-EIF3G and Myc-SRPX, respectively, were shown in input lysates and in anti-HA tag and anti-PELO immunoprecipitates but not in rabbit IgG-immunoprecipitates (Figure 4B and 4C). Collectively, these results clearly demonstrate that PELO forms protein complexes with HAX1, EIF3G and SRPX in HeLa cells.

### Co-localization of PELO and its interacting proteins at the cytoskeleton

**Figure 5:**
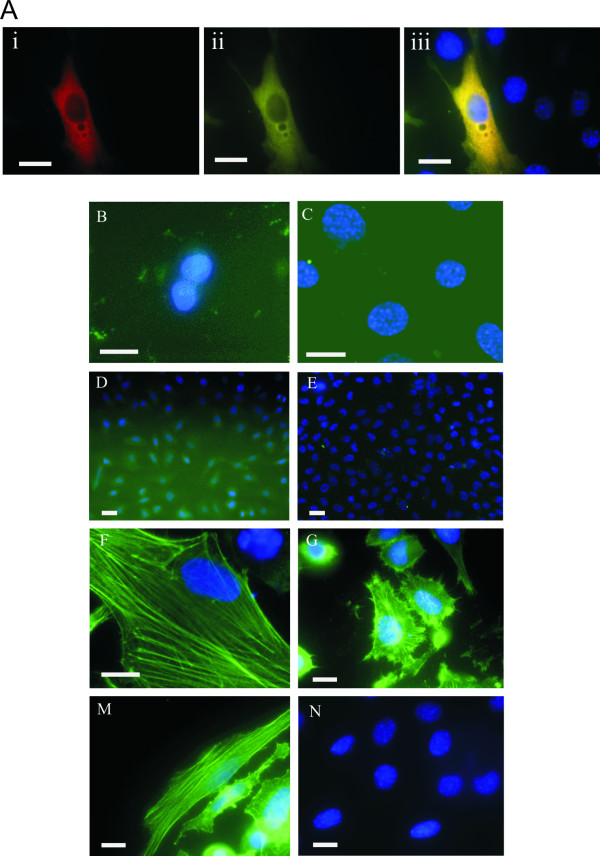
**Cellular localization of PELO-interacting proteins**. **A**, HeLa cells were transiently co-transfected with PELO-HA and Myc-HAX1 and immunostained with anti-HA (panel i) and anti-Myc (panel ii) antibodies. A co-localization of both proteins is mainly diffused in cytoplasm (panel iii). **B-N**, Bimolecular fluorescence complementation analysis (BiFC) revealed that protein complexes between PELO and HAX1, EIF3G or SRPX are localized at the cytoskeleton. **B-E**, Cells transfected with GFPN (B) and GFPC (C), PELO-GFPC (D) and HAX1-GFPN (E) alone did not show any fluorescence. **F-N**, HeLa cells were transiently co-transfected with PELO-GFPC and either HAX1-GFPN (F), EIF3G-GFPN (G), SRPX-GFPN (M) or MOS-GFPN (N) as control. Complementation of both non-fluorescent GFP halves resulted in functional fluorophores, which is diagnostic for stable interaction of the fusion proteins. The reconstituted GFP-fluorescence (BiFC) is shown in green and mainly localized at the cytoskeleton. Absence of GFP-fluorescence (N) indicates lack of BiFC. DNA staining is shown in blue. Scale bars = 20 μm.

Similar to the subcellular distribution of PELO, HAX1 was also reported to be localized to actin stress fibers [8]. Transiently co-transfected HeLa cells were immunofluorescent stained with anti-HA and anti-Myc antibodies to show whether PELO and its binding partners HAX1, EIF3G or SRPX are associated with the cellular cytoskeleton. PELO-HA and Myc-HAX1 were diffusely distributed in the cytoplasm of co-transfected cells (Figure 5A, panel i-iii). These results further support our finding that the overexpression of PELO in stable overexpressing cell lines disrupts the organization of the actin cytoskeleton.

To overcome the failure to determine the subcellular localization of the protein interaction in living cells and to further support the biochemical interactions of PELO with its putative binding partners, we performed bimolecular fluorescence complementation assays (BiFC). This approach is based on the complementation between two non-fluorescent fragments of EGFP when they are brought together by interaction of two proteins fused to each fragment. This assay is effective in determining the interaction of proteins by visualization of fluorescence on protein complexes in living cells [9,10]. For BiFC analysis, we generated an expression vector by fusing the full-length PELO cDNA in-frame to the C-terminal half of EGFP (PELO-GFPC), whereas the N-terminal half of EGFP was fused with HAX1 (HAX1-GFPN), EIF3G (EIF3G-GFPN) or SRPX (SRPX-GFPN). Cells transfected with either both empty vectors or with either PELO-GFPC or HAX1-GFPN alone did not show any fluorescence (Figure 5B-E). In contrast, cells co-transfected with PELO-GFPC and HAX1-GFPN displayed fluorescence, which was mainly accumulated at the cytoskeletal filaments (Figure 5F). Similar patterns of subcellular localization of fluorescent complementation were observed when the fusion proteins PELO-GFPC with either EIF3G-GFPN or SRPX-GFPN were expressed in HeLa cells (Figure 5G and 5M). In a control experiment, cells co-transfected with PELO-GFPC and MOS-GFPN containing the sequence of the MOS gene, which did not show to be an interacting-protein of PELO, did not reveal any fluorescent staining (Figure 5N). Taken together, these results demonstrate that PELO interacts with HAX1, EIF3G and SRPX, and the resulting protein complexes are associated with the cytoskeleton.

## Discussion

Current data on the consequences of PELO deficiency in *Drosophila *and mouse suggest that PELO is involved in the regulation of stem cell renewal [6,7]. In yeast Dom 34/PELO has been described to play a critical role in a new surveillance pathway termed No-Go decay [11,12]. This pathway clears cells from mRNAs that induce translational stalls through endonuclease cleavage that is thought to be mediated by Dom34/PELO and its interacting protein Hbs1. Dom34 and Hbs1 are related to the translation termination factor eRF1 and eRF3, respectively. Beside the role of eRF1 and eRF3 in translation, both proteins are involved in cytoskeleton organization in yeast [13]. Deletion of eRF3 disrupted actin cytoskeleton in most cells. In contrast, these effects were not observed in cell deficient for eRF1, a structural homolog PELO [13].

Following cell adhesion, cells rearrange their cytoskeleton and spread by increasing their contact with extracellular matrix, and through formation of actin stress fibers and focal adhesions [14-16]. Analyses of stable PELO-expressing cell lines revealed that cell lines showing higher PELO expression, which was correlated with reduced growth rates, spread much slower. These results suggest that expression of PELO at higher levels resulted in the disassembly of actin stress fibers and reduced cell spreading.

To gain more insight into the function of PELO, a cDNA library was screened by yeast two-hybrid assay for PELO-interacting proteins. This lead to the identification of three proteins that interact with PELO: multifunctional protein HAX1, the eukaryotic initiation factor EIF3G and the apoptosis-inducing tumor suppressor SRPX/RDS. The interactions of PELO with HAX1, EIF3G and SRPX in yeast were corroborated by three lines of evidences. All GST-PELO fusion proteins except those lacking the C-terminal domain of PELO extracted the recombinant Myc-HAX1, Myc-EIF3G and Myc-SRPX from extracts of transiently transfected HeLa cells suggesting that the C-terminal domain of PELO is responsible for specific binding with interacting proteins. PELO was specifically co-immunoprecipitated with HAX1, EIF3G and SRPX from cell extracts. Finally, BiFC analysis revealed that formed protein complexes are localized at the cytoskeleton of mammalian cells.

HAX1, a 34 kDa polypeptide, interacts with a heterogeneous group of proteins including the α-subunit of the heterodimeric G-protein Gα13 and cortactin, which are colocalized with HAX1 at the actin cytoskeleton. Overexpression of HAX1 in stably transfected cells reduces the formation of actin stress fibers [8], a phenotype which was also shown in PELO-overexpressing cell line. HAX1 is a multifunctional protein, active in different cellular compartments and involved in various cellular processes. Attention has been focused on its function in apoptosis and regulation of the actin cytoskeleton. However, it has been reported that HAX1 is an RNA-binding protein and may be has a regulatory role in stabilization and/or transport of mRNA [17,18].

EIF3G represents a subunit of the eukaryotic translation initiation factor 3 (eIF3). The eIF3 complex consists of at least 13 subunits and plays a central role in the pathway of protein translation initiation [19-21]. EIF3G has been reported to associate with the cytoskeletal protein 4.1R and with AIF that induce apoptosis through inhibition of protein synthesis [22,23].

A third PELO-interacting protein, SRPX, was originally isolated as a suppressor of v-src transformation [24,25]. Subsequent reports found that SRPX induces apoptosis via a novel ER-mediated pathway and suggest that this pathway might contribute to the suppression of tumor formation [26].

Various studies have shown that mRNA, polysomes and different components of the protein synthesis machinery, such as aminoacyl-tRNA, eukaryotic initiation factors and elongation factors are associated with the cytoskeleton and that this association may influence the transport and translation of mRNA [27,28]. Therefore, binding of PELO to cytoskeleton-associated proteins may facilitate PELO to detect and degrade aberrant mRNAs, at which the ribosome is stalled during translation.

PELO has an endonuclease activity that catalyzes the mRNA cleavage near the stall site of the ribosome and subsequent degradation [29]. However, mutations inactivating the proposed endonuclease domain do not affect No-Go decay (NGD). These results suggest that the proposed endonuclease activity of Dom34/PELO is not required for mRNA cleavage in NGD [30]. The interactions of PELO with components of the protein synthesis machinery and apoptosis raise the question whether PELO has a role in linking mRNA and protein synthesis to apoptosis. Several studies revealed an increase of mRNA degradation during cell death. Such increase of mRNA degradation seems to be correlated very well with the inhibition of protein synthesis [31,32]. Therefore, it is probable that mRNA decay makes a significant contribution to the reduction in global protein synthesis during apoptosis [33]. The link between mRNA decay and protein synthesis has been supported by the observation showing that the RNaseL regulates mitochondrial mRNA decay during apoptosis through its interaction with mitochondria initiation factor 2 (IF2 mt). This interaction brings RNaseL into close association with the mRNA, where it can act as an endonuclease [34]. These observations lead us to suggest that the interaction of PELO with EIF3G mediates the inhibition of protein synthesis during cell death by global mRNA decay or by inhibiting translation initiation.

## Conclusion

In summary, the experiments here have demonstrated that PELO is localized to actin cytoskeleton. Yeast two-hybrid, GST pull-down and coimmunoprecipitation studies revealed that PELO interacts with HAX1, EIF3G and SRPX. These results were further confirmed by BiFC assay showing that protein complexes resulting from the interactions between PELO and either HAX1, EIF3G or SRPX were mainly localized to cytoskeletal filaments.

## Methods

### Production of anti-PELO antibodies

Antibodies against the full-length of human PELO fused to GST (GST-PELO) were raised in rabbits (see below). Rabbits were initially injected with 1.0 mg of purified GST-PELO antigen mixed 1:1 with complete Freund's adjuvant followed by two boosts, at 3-weeks intervals, with 0.5 mg of antigen mixed 1:1 with incomplete Freund's adjuvant. The antibodies were affinity purified by absorption to the same fusion protein covalently coupled to HiTrap NHS-activated columns (Amersham Biosciences, Braunschweig, Germany) following the manufacturer's procedures.

### Cell fractionation

HeLa cells were washed twice with PBS, and scraped in 0.5 ml of lysis buffer (50 mM Tris-HCl, pH 7.5, 2 mM EDTA, 2 mM EGTA, 1 mM sodium vanadate, 1 mM dithiothreitol, 0.1% Triton X-100, protease inhibitor cocktail) on ice for 15 min. The cells were homogenized with a glass Dounce homogenizer. The nuclear fraction was collected by centrifugation at 600 × g for 20 min at 4°C. The supernatant was further centrifuged at 100 000 × g for 60 min to collect a cytoplasmic soluble supernatant and a plasma membrane pellet. The nuclear and plasma membrane pellet was extracted with RIPA lysis buffer (Santa Cruz Biotechnologie, Heidelberg, Germany). To obtain a cytoskeletal fraction, cells were scraped on ice in cytoskeleton-stabilizing buffer (50 mM NaCl, 10 mM Pipes, pH 6.8, 3 mM MgCl_2_, 0.5% Triton X-100, protease inhibitor cocktail), and centrifuged at 14000 × g at 4°C. The pellet was resuspended in cytoskeleton-stabilizing buffer containing 0.5% SDS.

### Yeast two-hybrid screening

To identify putative PELO-interacting proteins, human prostate cDNA library in pACT2 (BD Bioscience, Heidelberg, Germany) was screened using the pGBK7-PELO bait vector according to the manufacturer's instructions. Using sequential transformations, the bait vector and then the prey library were transformed into the AH109 strain. After the first transfection, the bait vector was characterized for lack of autoactivation by growing the PELO-transformants on plate lacking histidine and adenine. Double transformants were initially selected by growth on plates lacking histidine and adenine in the presence of 5 mM 3-amino-1,2,3-triazole (Sigma-Aldrich, Munich, Germany). From 1 × 10^7 ^transformants, 98 clones appeared after 5 days. These colonies were plated on plates lacking both histidine and adenine and containing β-galactosidase. Approximately 56 of those displayed clear activation of all three reporter genes, and 42 of these were selected for isolation of the prey plasmids and sequencing of the cDNA encoding the PELO-interacting protein.

### Expression of proteins in mammalian cells

For transient expression, human HeLa cells were grown for 24 h on 6-well plates (1.5 × 10^5 ^cells/well) prior to transfection with recombinant construct (2 μg/well) using Lipofectamin 2000 transfection reagent (Invitrogen, Karlsruhe, Germany) according to the manufacturer's procedures. Transfected cells were further cultured for 2 days prior to harvesting for co-immunoprecipitation studies. For stable expression, HepG2 cells were transfected either with pcDNA™ 3.1/Myc-His A(+) vector alone (Invitrogen) or with recombinant vector containing the full-length *PELO *cDNA, which was in-frame with the Myc tag. The cells were trypsinized 48 h after transfection, and cultured in medium containing 600 μg/ml of G418. After 2 weeks, single colonies were picked, cultured and the expression of Myc-tagged PELO was analysed by immunoblotting using Myc tag-specific monoclonal antibody (1:200 dilution, Santa Cruz Biotechnologie).

### Immunoprecipitation and Immunoblotting

The plasmid constructs Myc-HAX1, Myc-EIF3G and Myc-SRPX, which were used in co-immunoprecipitation experiments, were generated by amplification the cDNA fragments of HAX1, EIF3G und SRPX using the identified prey vectors as template and the sense primer Y2HF: 5'-TTC GAT GAT GAA GAT ACC CCA AAC-3' and antisense primer Y2HR: 5'-GTG AAC TTG CGG GGT TTT TCA GTA TCT AC-3'. The amplified PCR fragments were inserted into the *Eco*RI/*Xho*I sites of the pCMV-Myc vector (BD Bioscience). The PELO-HA construct was generated by insertion of cDNA fragment of full-length human PELO into the *Eco*RI/*Xho*I sites of the pCMV-HA vector (BD Bioscience), which had a HA epitope linked to the N-terminus of PELO protein. HeLa cells were transiently co-transfected with PELO-HA and either of the Myc-HAX1, Myc-EIF3G or Myc-SRPX constructs. For co-immunopreciptation, 500 μl of whole cell lysates were firstly pre-cleared by incubation with 50 μl protein A/G-agarose beads (Roche, Mannheim, Germany) for 3 h. The pre-cleared lysates were then incubated with either 10 μl mouse monoclonal anti-Myc (Santa Cruz Biotechnology), 10 μl rabbit polyclonal anti-HA (BD Biosciences) or 4 μl polyclonal anti-PELO antibodies for 2 h at 4°C. In control assays, lysates were subjected to immunoprecipition using 4 μl of mouse IgG. The immunoprecipitates were recovered by incubating the mixture overnight at 4°C with 50 μl protein A/G-agarose beads. After extensive washing of the beads, bound proteins were separated by SDS-PAGE and transferred to nitrocellulose membrane Hybond-C (Amersham Biosciences). Membranes were blocked for 1 h in 5% non-fat dry milk in PBS containing 0.2% Tween20 and then incubated overnight at 4°C with either anti-Myc, anti-HA or anti-PELO antibodies (1:500 dilution). Bound antibodies were then visualized by secondary incubation with goat anti-rabbit IgG horseradish peroxidase conjugate or goat anti-mouse IgG horseradish peroxidase conjugate (Sigma-Aldrich) followed by SuperSignal^® ^West Pico Chemiluminescent Substrate (Pierce, USA).

### GST pull-down assay

For generation of glutathione S-transferase-PELO and truncated fusion constructs, cDNA fragments encoding full-length human Pelo (GST-1, 2-385 aa), and truncated Pelo - GST-2 (136-385 aa), GST-3 (2-371 aa) and GST-4 (2-268 aa) were amplified by PCR using human *Pelo *cDNA as template and appropriate primers containing restriction site tags. Oligonucleotide sequences are given in Additional file 1. The amplified fragments were cloned in-frame into *Eco*RI/*Xho*I sites of the pET41a(+) vector (Novagen, Darmstadt, Germany). B121(DE3)pLysS bacteria were transformed with either empty GST vector (GST-0) or GST-PELO fusion vectors and grown at 37°C to log phase (A_600 _of 0.6). Expression of fusion protein was induced by growing the cells at 30°C for 4 h in the presence of 100 μM 1-thio-β-D-galactopyranoside. GST-PELO fusion proteins were purified using the GST-bind™ Purification Kit (Novagen, Darmstadt, Germany). HeLa cell protein extracts (500 mg protein) were incubated with 50 μl (100 μg) of individual fusion protein or GST alone in the GST binding buffer for 3 h at 4°C. After centrifugation, pellets were washed 3 times with lysis buffer B, boiled in sample buffer, followed by SDS-PAGE and Western blot analysis.

100 μl of GST-bind-resin were transferred to a clean 1.5 ml microcentrifuge tube and washed 3 times with 250 μl of 1× GST Bind/Wash buffer. The beads were resuspended in 50 μl 1× GST Bind/Wash buffer and incubated with 50 μl (100 μg) of individual GST fusion protein or GST alone for 1 h at RT on a rocking platform. Beads were washed 3 times with 250 μl of 1× GST Bind/Wash buffer and 100 μl of HeLa cell lysates (300 - 500 μg protein) were added to the reaction tube and the mixture was incubated for 2 h at 4°C on a rocking platform. The beads were then washed 3 times with 250 μl of lysis buffer B. Finally, proteins binding to the beads were resuspended with NuPAGE SDS sample buffer, separated by SDS-PAGE and analyzed by Western blotting using anti-c-Myc primary antibodies (dilution 1:200) and goat anti-mouse IgG conjugated with horseradish peroxidase secondary antibodies. Immunoreactive polypeptides were then visualized by the SuperSignal^® ^West Pico Chemiluminescent Substrate as previously described.

### Spreading, proliferation and soft agar assays

HepG2 cells stably transfected with empty pcDNA3A-Myc or pcDNA-PELO plasmids were seeded at a density of 2 × 10^4 ^cell/well onto 96-well plates that were pre-coated with 10 μg/ml fibronectin (Sigma-Aldrich). Cells were allowed to spread for 60 min at 37°C in growth medium. Non-adherent cells were removed by two washes with pre-warmed PBS. The percentage of spreaded cells was evaluated 30 min and 120 min after seeding by counting the number of non-spread cells under the microscope (Olympus BX60, Hamburg, Germany). The non-spread cells were defined as round cells, whereas spread cells were defined as those that lacked a rounded shape and had extended membrane protrusions [35]. To obtain an index of cell spreading, adherent cells were photographed with a digital camera. According to their distinct differences in surface area and morphology, the cells were divided into two groups: small round cells showing no obvious sign of spreading (group 1), and flat cells in the process of spreading or already well spread (group 2). Monitoring the proportion of cells in group 1 to total cells at a given time point after seeding serves as a measure of spreading velocity. At least 100 cells per well were counted in three independent experiments.

For cell proliferation assay, stably transfected HepG2 cells with empty pcDNA3A-Myc or pcDNA-PELO plasmids were seeded in triplicate in 24-well plates (0.25 × 10^5 ^cells per well) and cultivated for 5 days. Every day, cells were washed, trypsinized and proliferation of cells was evaluated by counting the number of cells under the microscope using a Neubauer counting chamber.

For soft agar colony formation assay, single cells of stably transfected HepG2 cell were resuspended in semi-solid medium containing 0.35% Bacto-agarose supplemented with 20% FBS, 750 μg/ml G418 and 0.8% agarose. This cell suspension containing 0.2 × 10^4 ^cells/well was immediately plated into 6-well plates coated with 0.3% agar in cell culture medium (2 ml per well) and cultured at 37°C with 5% CO_2_. After 3 weeks, the top layer of the culture was stained with 0.1% crystal violet for 1 h. The culture was analyzed in triplicate and colonies larger than 100 μm in diameter were counted.

### Immunofluorescence

Cells were grown on chamber slide overnight, fixed with 4% paraformaldehyde in PBS for 15 min. Subsequently, cells were washed with PBS, preincubated with 0.2% Triton X-100 in PBS for 30 min and blocked with 10% goat serum in PBS for 1 h. Cells were incubated overnight at 4°C with 100 μl of primary antibody diluted in 1% goat serum in PBS. Primary antibodies were as followed: a 1:200 dilution of anti-PELO antibody, a 1:200 of anti-Myc and anti-HA antibodies. Cells were washed with PBS and then incubated with a 1:500 dilution of the appropriate FITC-coupled secondary antibody (Sigma-Aldrich) in 1% goat serum in PBS for 2 h. Cells were washed and then incubated with 1:100 dilution of FITC-phalloidin (Sigma-Aldrich) in PBS for 20 min. Cells were mounted with Vectashield mounting reagent (Vector, Burlingame, USA) prior to fluorescence microscopy (Olympus, Hamburg, Germany).

### Bimolecular fluorescence complementation (BiFC) assay

The pEGFP-N1 (BD Bioscience) and pQM-Ntag/B (Abcam, UK) vectors were used as start plasmids to construct the BiFC vector FPCA-V1 and FPCA-V2, respectively. To generate the FPCA-V2 and FPCA-V1, fragments containing the coding sequence for the residues 1-157 or the residues 158-239 of the EGFP fragment were amplified by PCR and inserted into the backbone of the pQM-Ntag/B and pEGFP-N1 vectors, respectively. The full-length cDNA fragment of human *Pelo *(1-385 aa) was amplified and cloned into the *Xho*I/*Bam*HI-digested FPCA-V1 to generate PELO-GFPC construct. The cloned cDNAs of HAX1 (178-279 aa), EIF3G (1-320 aa) and SRPX (265-464 aa) were amplified by PCR and inserted into *Hind*III/*Bgl*II-digested FPCA-V2 to generate HAX1-GFPN, EIF3G-GFPN and SRPX-GFPN constructs, respectively. Primer sequences used for generation a cDNA fragments are presented in Additional file 1. HeLa cells grown on chamber slides were transiently co-transfected with PELO-GFPC and either HAX1-GFPN, EIF3G-GFPN or SRPX-GFPN constructs. Transfected cells were grown at 37°C for 24 h, incubated for 2 h at 30°C and then fixed in 4% paraformaldehyde in PBS for 10 min. Single transfection of the plasmids used for BiFC (PELO-GFPC and HAX1-GFPN) did not produce any fluorescence.

## Authors' contributions

AK, OBT, NK participated in GST pull-down, immunoprecipitation and BiFC experiments. LE carried out the yeast two-hybrid screening. BB carried out the generation of the anti-PELO antibody and immunoassays. PB participated in the construction of prostate cDNA library. SHF generated the BiFC vectors. IA and WE conceived of the study, and participated in its design and coordination and helped to draft the manuscript. All authors read and approved the final manuscript.

## Supplementary Material

Additional file 1 **Oligonucleotide primers**. Sequences of oligonucleotide primers used for amplification of cloned cDNA fragments. Click here for file
