# Supplementary material for: Pelota interacts with HAX1, EIF3G and SRPX and the resulting protein complexes are associated with the actin cytoskeleton
Source: BMC Cell Biol. 2010 Apr 20;11:28. doi: 10.1186/1471-2121-11-28 (PMC2867792; doi:10.1186/1471-2121-11-28)
Supplement: Additional file 1 — Oligonucleotide primers. Sequences of oligonucleotide primers used for amplification of cloned cDNA fragments. [file 1471-2121-11-28-S1.DOC]

**Additional file 1**

**Oligonucleotide primers.** Sequences of oligonucleotide primers used for amplification of cloned cDNA fragments

| Vector | 5´Primer | 3´Primer |
| --- | --- | --- |
| pGBK7-PELO | CCCGAATTCAAGCTCGTGAGGAAGAAC ATC | ACCGTCGACTCCTCTTCAGAACTGGAA TC |
| PELO-HA | CCCGAATTCAGCTCGTGAGGAAGAACATC | ACCCTCGAGTTAATCCTCTTCAGAACTGGAATC |
| PELO-Myc | GTGGAATTCTCCTTGGCCATGAAGCTCGTG | AGACTCGAGATCCTCTTCAGAACTGGAATCACC |
| GST-1 | GAATTCAAGCTCGTGAGGAAGGATATCGAGA | CTCGAGAATCCTCTTCAGAACTTGAATC |
| GST-2 | GAATTCGATGTGGCAGCTGTGGTCATG | CTCGAGAATCCTCTTCAGAACTTGAATC |
| GST-3 | GAATTCAAGCTCGTGAGGAAGGATATCGAGA | CTCGAGAAAGTTCTGGCACTGGGAAGC |
| GST-4 | GAATTCAAGCTCGTGAGGAAGGATATCGAGA | CTCGAGTGTGTGAAAGGCGGCTCGCTAC |
| PELO-GFPC | GCCCTCGAGATGAAGCTCGTGAGGAAGAACATC | TCCTGGATCCTCCTCTTCAGAACTGGAATCACC |
| HAX1-GFPN | AGGCAAGCTTATCCTAGAACCAGAGAGGACAATG | GGGAGATCTTTAACAAGGCTACCGGGACCGG |
| EIF3G-GFPN | AGGCAAGCTTTGCCTACTGGAGACTTCGATTCG | GGGAGATCTCTAGTTGGTGGACGGCTTGGCCCAC |
| SRPX-GFPN | AGGCAAGCTTTCAATGCCCCAGAGAATGGTTAC | GGGAGATCTTCAGGTGTTACAGTTCTGGCTCA |
